# Analysis of Risk Factors Affecting Postoperative Success Rate and Postoperative Pain After Orthodontic Microimplant Nail Implantation and Improvement of Treatment Strategy

**DOI:** 10.3290/j.ohpd.c_2349

**Published:** 2025-11-26

**Authors:** Yang Liu

**Affiliations:** a Yang Liu Orthodontist, Department of Stomatology, Qingdao West Coast New Area Central Hospital, Qingdao, Shandong 266555, China. Study design and concept, conducted the study, wrote and reviewed the manuscript.

**Keywords:** insertion torque, orthodontic microimplants, personalised management, postoperative pain, risk factors

## Abstract

**Purpose:**

This study aimed to investigate a range of patient- and procedure-related factors influencing the success rate and postoperative pain of orthodontic microimplants, and propose potential personalised management strategies based on a large, single-centre dataset.

**Methods and Materials:**

A total of 853 participants (1,277 microimplants) were included in this single-centre, retrospective investigation. Clinical and demographic information were collected. The primary outcome was implant-level failure within 6 months (yes/no), with success defined as stable retention and function for ≥6 months without severe inflammation or reinsertion. Postoperative pain was assessed at 6 h, 24 h, and 7 days using the visual analogue scale (VAS). Analgesic usage and soft-tissue inflammation were recorded as secondary outcomes. Microimplant success was defined by stable retention over at least 6 months, absence of severe inflammation, and no requirement for reinsertion. Multivariable logistic regression and Cox proportional hazards models were used to identify independent predictors of microimplant failure over time. In addition, a random forest model was employed to rank risk-factor importance.

**Results:**

Overall, 1,089 microimplants (90.8%) remained stable throughout the follow-up period, with a higher failure rate observed among older adults, smokers, and patients with lower cortical bone thickness (P <0.05). High insertion torque (>15 Ncm) significantly increased the odds of postoperative discomfort (OR = 1.85, 95% CI 1.30–2.65). Operator experience also correlated with lower failure and pain scores (P <0.01). The random forest analysis highlighted bone density, insertion torque, and smoking as the top three predictors.

**Conclusions:**

Incorporating individualised strategies can improve clinical outcomes. Controlling insertion torque, accounting for cortical thickness, counselling smokers, and allocating senior operators are actionable steps to improve stability and patient comfort.

Orthodontic anchorage techniques have evolved significantly over time, with traditional methods such as headgear and transpalatal arches being widely used to provide the necessary support for tooth movement. However, these methods often require patient compliance and could be cumbersome or uncomfortable. The introduction of microimplants, also known as mini-implants or temporary anchorage devices (TADs), has revolutionised orthodontic treatment by offering a more reliable and patient-friendly solution for temporary anchorage. These small, screw-like devices are strategically placed in the jawbone, providing stable anchor points that allow for precise tooth movement without affecting adjacent teeth.^27,41^ Microimplants offer several advantages over traditional methods, including ease of insertion and removal, minimal patient discomfort, and the ability to apply immediate force, which enhances treatment efficiency.^1,41^ They are particularly beneficial in cases requiring absolute anchorage, such as severe malocclusions and impacted teeth.^41^

The current use of orthodontic microimplants is widespread, with reported success rates ranging from 80% to 95%.^27,42^ The use of surgical guides has been shown to significantly improve the success rate of infrazygomatic mini-implants.^42^ The global acceptance of microimplants is attributed to their versatility and effectiveness in various orthodontic procedures.^41^ Additionally, innovations such as larger diameter and shorter length designs have been found to enhance mechanical stability.^11^ The integration of bioactive coatings, such as those enriched with silver nanoparticles, has also been explored to reduce the risk of infection and improve implant stability.^33^ Overall, the high success rates and broad clinical applications of microimplants underscore their role as a cornerstone in modern orthodontic practice.^17,28^ In parallel, contemporary periodontal literature emphasises that orthodontic biomechanics must be planned within a periodontally healthy or well-controlled environment. This includes structured care pathways for patients with active or treated periodontitis and the role of supportive periodontal care during active orthodontic therapy.^4,5^

The success of microimplants in dental procedures is influenced by several risk factors, including bone density, insertion torque, patient age, smoking habits, and operator experience. Compromised bone structures can complicate implant success due to fragile or insufficient bone volume.^14^ Improper torque potentially leading to implant failure or fractures, especially in regions subjected to high masticatory forces.^16^ Patient age and smoking have also been associated with varying success rates, although findings are sometimes conflicting or inconclusive, particularly regarding the impact of age on osseointegration.^22^ Operator experience is crucial, as it influences the precision of implant placement and the management of potential complications.^38^ Postoperative pain is a critical patient-centred outcome, with typical pain levels after insertion affecting patient compliance and overall treatment satisfaction. However, there is limited data on how specific risk factors correlate with pain severity or duration.^13^ Many studies addressing these issues are limited by being single-centre with small sample sizes.^13^ Furthermore, there is a notable gap in comprehensive studies linking success rates and postoperative pain in real-world clinical settings.^13,16^ The biological coupling between inflammation and force-induced bone remodelling, providing a mechanistic context for torque and bone-related risks, and suggests nonsurgical approaches that may modulate tissue responses during accelerated tooth movement. Interdisciplinary strategies for gingivalmargin optimisation in reduced periodontium complement risk-adapted placement and aftercare.^9,18,24^

The present study draws upon a large, single-centre cohort to address these gaps by systematically documenting a wide range of potential risk factors and evaluating their association with both microimplant stability and postoperative pain. Through stratified analyses and robust statistical models, this work aims not only to clarify which clinical and demographic variables most strongly predict implant success or failure over time but also to determine how these same variables influence postoperative discomfort, thereby offering new insights to guide personalised treatment strategies in orthodontic practice. This retrospective, singlecentre observational study had three prespecified objectives at the implant level: (1) to identify independent risk factors for failure within 6 months among orthodontic microimplants; (2) to describe the postoperative pain trajectory and to evaluate factors associated with higher pain and analgesic use; (3) to provide a variableimportance ranking using a randomforest model and to perform timetofailure analyses when data permitted.

## METHODS AND MATERIALS

## Study Design and Setting

We conducted a retrospective, singlecentre observational study. This retrospective observational study was conducted at Qingdao West Coast New Area Central Hospital. All data were retrieved from electronic health records and paper charts for patients treated between January 2017 and December 2022. Prior to data collection, the study protocol was submitted to and approved by the ethical committee at Qingdao West Coast New Area Central Hospital. All patient-related data were de-identified and analysed under strict confidentiality in compliance with the Declaration of Helsinki. Written informed consent was obtained from all participants.

## Participant Selection

**Inclusion criteria:** (1) patients aged ≥12 years who underwent orthodontic microimplant placement as part of their orthodontic treatment; (2) at least one microimplant placed for anchorage or auxiliary orthodontic mechanics; (3), a minimum 6-month follow-up after implant insertion (unless failure occurred earlier); (4), availability of key clinical information, including demographic data, implant details, postoperative records, and pain assessment data.
**Exclusion criteria:** (1) missing or incomplete records preventing confirmation of success/failure outcomes or postoperative pain scores; (2) patients with uncontrolled systemic diseases (eg, advanced cancer, uncontrolled diabetes) that severely compromise bone healing; (3), microimplants placed but never loaded or used in orthodontic mechanics; (4), implants placed outside of the alveolar bone (eg, specific palatal implants not meeting the same success criteria).

### Sample size calculation and participant flow

As this was a retrospective study, we initially planned to include all consecutive patients meeting the inclusion criteria during the specified time window. No formal a priori sample size calculation was performed beforehand. Instead, we employed the following considerations:

**Historical data:** Previous single-centre investigations on orthodontic microimplants often ranged from 100 to 500 implants, limiting statistical power to detect small-to-moderate effect sizes in risk-factor analysis.
**Anticipated failure rate:** The literature suggests failure rates for orthodontic microimplants of approximately 5–15%. We aimed to capture a sufficient number of events (failures) to reliably assess multiple risk factors in a logistic regression model.
**Rule of thumb for multivariate analyses:** Common guidelines suggest having at least 10 outcome events per predictor variable in a logistic regression model.20 Given our intent to analyse multiple covariates (eg, demographics, insertion torque, bone density, operator experience), a larger sample would ensure more stable estimates.
**Post-hoc,** we verified that the final number of 853 participants and 1,277 microimplants was sufficient to detect clinically meaningful differences, given the observed ~9–10% failure rate. This sample also allowed for subgroup analyses (eg, smokers vs non-smokers).
**Posthoc power and detectable effect sizes:** Using two-sided α = 0.05 and accounting for within-patient clustering (1,277 implants from 853 patients; mean cluster size ≈1.50) with an intrapatient ICC of 0.05 (effective N≈1,246), we estimated observed power for the key binary contrasts and the minimum detectable effect sizes. For smoking, 252 vs 1,025 implants with failure 16.7% vs 7.5% yielded 95.7% power. For insertion torque > 15 N·cm, 270 vs 1,007 implants with failure 22.2% vs 5.9% yielded >99.99% power. For operator experience (resident vs senior), 377 vs 900 implants with failure 13.0% vs 7.8% yielded 76.4% power. At the overall failure rate of 9.3%, the study provides 80% power to detect odds ratios of ≥1.91 (≈20% prevalence) and ≥1.75 (≈30% prevalence), and 90% power for ≥2.10 and ≥1.90, respectively. These quantitative estimates support adequate power for our primary objectives.

### Data collection

Trained research assistants extracted demographic information (age, sex, BMI, smoking status, relevant systemic conditions) from the hospital’s EHR. Smoking status was categorised as current, former, or never smoker. BMI was calculated if both height and weight data were available.

#### Implant details and surgical factors

Details on each microimplant – including brand/type, surface coating, length, and diameter – were recorded from operative notes. Surgical variables such as pre-drilling protocol, insertion torque (if documented in the chart or torque controller), and operator experience level (senior orthodontist vs. resident) were noted. Implant location was categorised as maxilla or mandible, and further subdivided into anterior or posterior regions based on the tooth site.

#### Conebeam computed tomography (CBCT) acquisition and quantitative measurements

For a subset of cases with a CBCT obtained as part of routine clinical care at the time of treatment planning, we quantified local bone characteristics at the microimplant insertion site. Using multiplanar reconstructions aligned to the alveolar process, assessors identified the slice passing through the eventual insertion point and measured: (1) cortical bone thickness (mm) of the alveolar cortical plate at the insertion site; and (2) bone density in the adjacent trabecular compartment using the scanner-reported Hounsfieldunit (HU) scale. Because absolute HU values from CBCT can vary across devices and acquisition settings, we prespecified three density categories for analysis: low (<600 HU), intermediate (600–1,000 HU), and high (>1,000 HU). Cortical thickness was analysed as a continuous variable, while density entered models as a three-level factor.

## Outcome Measures

### Primary outcome

The primary outcome variable was coded as a binary indicator of failure within 6 months after placement (yes/no). Failure was recorded if the microimplant loosened, required removal or replacement, or exhibited persistent soft-tissue inflammation leading to removal within 6 months. Success required stable retention and function for ≥6 months without severe inflammation and without reinsertion. For reporting, early failure was defined as <1 month and late failure as 1–6 months. Both were analysed as failures in the primary multivariable logistic regression. For supportive survival analyses, timetofailure was measured from insertion to the failure date.

### Secondary outcome

Postoperative pain was primarily assessed using a visual analogue scale (VAS) of 0–10, documented at approximately 6 h, 24 h, and 7 days post-insertion. Analgesic use (type, dosage, frequency) was extracted from prescription records when available. Additional time points were aggregated if consistently documented.

### Statistical analysis

All data were entered into a secure spreadsheet and cross-checked by two independent researchers. Discrepancies were resolved through discussion with a senior investigator. Continuous variables were summarised as means ± standard deviations (SD) or medians (interquartile ranges, IQR) based on normality. Categorical variables were summarised as frequencies (percentages). Differences between successful vs. failed implants were evaluated using t-tests or Mann–Whitney U tests for continuous variables, and Chi-square or Fisher’s exact tests for categorical variables. Significance was set at P <0.05.

The primary analysis modelled the binary failurewithin6months of outcome at the implant level using multivariable logistic regression. Supportive analyses used timetofailure with Kaplan–Meier/Cox methods. In descriptive tables, proportions are reported with Wilson 95% confidence intervals. Univariate comparisons include unadjusted odds ratios (binary predictors, Wald 95% CIs) or mean differences (continuous predictors, Welch 95% CIs). For CBCT variables, cortical thickness was modelled as a continuous covariate (mm) and bone density as a three-level categorical factor (<600, 600–1000, >1,000 HU). Analyses involving CBCT were performed on the CBCT subset with completecase evaluation. To guard against overfitting, we verified that the final multivariable logistic model achieved an eventsperpredictorparameter ratio well above classical heuristics (EPP ≈ 23.8), in line with contemporary guidance.^5,21,26,36,37^

### Subgroup analyses

We prespecified subgroup analyses by smoking status (current vs noncurrent) and operator experience (resident vs senior) based on (i) biological/procedural plausibility that these axes could modify the effects of torque and bone quality on failure; (ii) clinical actionability (cessation support; operator allocation/supervision); and (iii) adequate, reasonably balanced sample sizes in both strata (smokers 252 vs nonsmokers 1,025 implants; residents 377 vs seniors 900). In contrast, age and insertion site were handled as adjustment covariates (age modelled continuously; site as maxilla/mandible) to preserve power given the total number of failures (n = 119) and to avoid redundancy with cortical thickness/bone density and insertion torque, which already capture site-related biology. This approach limited multiplicity and reduced sparsedata bias while retaining interpretability.

Missing data were primarily handled by complete-case analysis. In cases with >10% missingness for key variables, multiple imputation was explored to test the robustness of findings.

#### RESULTS

A total of 853 participants were included in this retrospective analysis (Table 1). Their mean age was 25.6 ± 8.4 years, with just over half (61.4%) being female. Most patients (87.9%) were under 40 years of age, and 23.4% reported being current smokers. The mean BMI was 23.1 ± 3.6 kg/m^2^, and 5.0% of participants had documented systemic conditions such as diabetes. The average duration of orthodontic treatment was 18.2 ± 5.1 months.

**Table 1:** Baseline characteristics of the participants

	Value
**Age, years**	25.6 ± 8.4
**Age category**	
<20	350 (41.0%)
20–39	400 (46.9%)
≥40	103 (12.1%)
**Sex**	
Male	329 (38.6%)
Female	524 (61.4%)
**BMI (kg/m** ^2^ **)**	23.1 ± 3.6
**Smoking Status**	
Current smoker	200 (23.4%)
Former smoker	120 (14.1%)
Never smoker	533 (62.5%)
**Systemic conditions (eg, diabetes)**	43 (5.0%)
**Duration of orthodontic treatment (months)**	18.2 ± 5.1


Across the 853 participants, a total of 1,277 microimplants were placed (Table 2). Brand A devices accounted for 54.8% of the implants, while 45.2% were Brand B. On average, the microimplants measured 6.0 ± 0.5 mm in length and 1.4 ± 0.1 mm in diameter, with 70.0% having a coated surface treatment. Most implants (62.7%) were inserted with a torque of 11–15 Ncm, while 21.7% required a torque above 15 Ncm. A pilot hole protocol was documented in approximately 78.3% of cases. Senior orthodontists conducted the majority of placements (70.5%), predominantly in the maxilla (70.5%).

**Table 2:** Implant-related and surgical factors

Variable	Value or distribution
**Length (mm),** Mean ± SD	6.0 ± 0.5
**Diameter (mm),** Mean ± SD	1.4 ± 0.1
**Surface treatment, n (%)**	
**Coated**	894 (70.0%)
**Non-coated**	383 (30.0%)
**Insertion torque (Ncm), n (%)**	
≤10	200 (15.7%)
11–15	800 (62.7%)
>15	277 (21.7%)
**Operator experience, n (%)**	
Senior orthodontist	900 (70.5%)
Resident	377 (29.5%)
**Insertion site, n (%)**	
Maxilla	900 (70.5%)
Mandible	377 (29.5%)
**Number of Implants per patient, n (%)**	
1 implant	400 (46.9%)
2 implants	300 (35.2%)
≥3 implants	153 (17.9%)


In a subset of cases with available CBCT data (Table 3), the mean cortical bone thickness was 1.4 ± 0.3 mm in the maxilla and 2.0 ± 0.4 mm in the mandible. Average bone density measured 850 ± 200 HU. Categorically, 23.5% of implants in this subset were placed in areas classified as ‘high density’ (>1000 HU), whereas 21.7% were considered ‘low density’ (<600 HU).

**Table 3:** Radiographic findings

	Mean ± SD
**Cortical bone thickness (mm)**	
Maxilla	1.4 ± 0.3
Mandible	2.0 ± 0.4
**Bone density (HU)**	850 ± 200
CBCT measurements were extracted from clinically indicated scans at treatment planning; no additional imaging was obtained for research. Because absolute CBCT HU values are devicedependent, bone density was analysed in predefined categories: <600, 600–1000, >1,000 HU.

Of the 1,277 microimplants evaluated, 1,158 (90.7%) remained successful throughout at least 6 months of follow-up (Table 4). Early failures (within one month) were observed in 4.3% of implants, while late failures (≥1 month) accounted for 5.0%. The mean time to failure was 3.2 ± 1.1 months (range, 0.5–6.0). Most failures were associated with either persistent inflammation or implant loosening.

**Table 4:** Overall success and failure rates of orthodontic microimplants

Outcome	n (%)	95% CI
**Success (Total)**	1,158 (90.7%)	89.0–92.2
Early success	1,050 (82.2%)	80.0–84.2
Late success	108 (8.5%)	7.1–10.1
**Failure (Total)**	119 (9.3%)	7.8–11.0
Early failure	55 (4.3%)	3.3–5.6
Late failure	64 (5.0%)	3.9–6.3
**Mean time to failure (months)**	3.2 ± 1.1 (range 0.5–6.0)	
Primary outcome was implant‑level failure within 6 months (yes/no); early failure <1 month, late failure 1–6 months. Success required stability for at least 6 months without severe inflammation or reinsertion.

Postoperative discomfort was assessed via VAS at three intervals (Table 5). The highest mean pain score (3.5 ± 1.7) occurred at 6 h post-placement, decreasing to 2.1 ± 1.3 at 24 h, and further to 0.9 ± 0.7 by day 7. Nearly half of the patients (46.9%) used analgesics at least once in the first week after implantation. Pain levels generally correlated with insertion torque and operator experience in the descriptive data, although formal testing is reported below.

**Table 5:** Postoperative pain scores (VAS) at different time points

Time point	Number (Implants/Patients)	Mean ± SD	Median (IQR)	Range
**6 h**	1,277/853	3.5 ± 1.7	3 (2–5)	0–8
**24 h**	1,277/853	2.1 ± 1.3	2 (1–3)	0–6
**7 days**	1,277/853	0.9 ± 0.7	1 (0–1)	0–4
**Analgesic usage**	853	400 (46.9%)	–	–


Univariate comparisons (Table 6) revealed that several factors were significantly associated with implant failure. Patients experiencing failure were older on average (28.1 ± 9.2 vs 24.9 ± 7.9 years, P <0.001) and more likely to be current smokers (35.3% vs 18.1%, P <0.001). Cortical bone thickness was lower in failed implants (1.3 ± 0.4 mm) compared to successful ones (1.6 ± 0.5 mm, P <0.001). Furthermore, a notably higher proportion of failed implants (50.4%) required insertion torque greater than 15 Ncm (P <0.001). Implants placed by senior orthodontists were less likely to fail than those placed by residents (58.8% vs. 71.7% success, P = 0.004).

**Table 6:** Univariate analysis of factors associated with microimplant failure

Factor	Success (n = 1,158)	Failure (n = 119)	95% CI	P value
**Age (per 10-year increase)**	24.9 ± 7.9	28.1 ± 9.2	1.49–4.91	<0.001
**Smoking (Yes vs No)**	210 (18.1%)	42 (35.3%)	1.64–3.69	<0.001
**Cortical thickness (mm)**	1.6 ± 0.5	1.3 ± 0.4	−0.38 to −0.22	<0.001
**Insertion torque >15 Ncm**	210 (18.1%)	60 (50.4%)	3.11–6.78	<0.001
**Operator experience (Senior)**	830 (71.7%)	70 (58.8%)	0.38–0.83	0.004
For binary factors, unadjusted odds ratios (OR) with Wald 95% CIs were computed from 2×2 counts. For continuous factors, Welch’s 95% CI for the mean difference (Failure − Success) is reported.

When significant covariates were entered into a multivariable logistic regression model (Table 7), age (per 10-year increment; OR = 1.29, 95% CI 1.09–1.53, P = 0.003), smoking status (OR = 2.21, 95% CI 1.49–3.27, P <0.001), lower cortical thickness (per 0.5 mm increase; OR = 0.68, 95% CI 0.56–0.84, P <0.001), and high insertion torque (>15 Ncm; OR = 2.78, 95% CI 1.98–3.92, P <0.001) independently predicted failure. Implants placed by senior orthodontists showed a significantly lower odds of failure (OR = 0.61, 95% CI 0.42–0.90, P = 0.015).

**Table 7:** Multivariable logistic regression predicting microimplant failure

Variable	Adjusted OR (95% CI)	P value
**Age** (per 10-year increase)	1.29 (1.09–1.53)	0.003
**Smoking** (Yes vs No)	2.21 (1.49–3.27)	<0.001
**Cortical thickness** (mm)	0.68 (0.56–0.84)	<0.001
**Insertion torque** >15 Ncm	2.78 (1.98–3.92)	<0.001
**Operator experience** (Senior vs Resident)	0.61 (0.42–0.90)	0.015


A subgroup analysis (Table 8) further underscored the influence of smoking on outcome: among smokers (n = 252 implants), the failure rate was 16.7%, nearly double that of non-smokers (7.5%, P <0.001). This effect remained robust even after controlling for other variables, suggesting that smoking status amplifies the impact of other risk factors such as cortical thickness or high insertion torque.

**Table 8:** Subgroup analysis by smoking status

Subgroup	Total implants (n)	Success (n, %)	Failure (n, %)	P value
**Smokers**	252	210 (83.3%)	42 (16.7%)	<0.001
**Non-smokers**	1,025	948 (92.5%)	77 (7.5%)
Subgroups were pre-specified for smoking (current vs non-current) and operator experience (resident vs senior) on clinical/biological grounds and samplesize balance. Age and insertion site were treated as adjustment covariates to minimise multiplicity and sparsedata bias.

#### DISCUSSION

In this retrospective, single-centre analysis, orthodontic microimplants achieved an overall success rate of approximately 90%, with older age, current smoking status, and high insertion torque (>15 Ncm) emerging as significant and independent risk factors for failure. Operator experience also showed a meaningful protective effect, suggesting that both clinical expertise and careful torque control are critical to long-term stability.

In the present study, our overall success rate of 90.7% aligns well with previously reported ranges for orthodontic microimplants (80–95%), though some studies have noted even higher rates when using individualised surgical guides.^2,15,42^ The slight variation may result from differences in implant design, operator skill, and variations in patient populations, including alveolar bone density and cortical thickness.^3,15,35^ Consistent with existing evidence, our findings highlighted factors like low cortical thickness, high insertion torque (>15 Ncm), and smoking as significant predictors of failure.^7,12,15^ Smoking emerged as a particularly potent risk factor, nearly doubling the odds of implant failure compared to non-smokers.^25^ Postoperative pain peaked at 6 h and declined steadily thereafter, which agrees with prior research indicating that early intervention in the first 24 h is critical.^10,19^ Although the majority of patients did not require strong analgesics, our results corroborate the literature on the multifactorial nature of pain, influenced by surgical trauma, psychological factors, and individual pain thresholds.^29,40^

Our findings align with the current periodontalorthodontic consensus that controlled biomechanics in an inflammation-free milieu are central to stability. Reviews from *Periodontology 2000* emphasise that structured protocols for delivering orthodontic treatment in periodontitis patients, including supportive periodontal care at regular intervals, and mechanistic links between inflammatory signalling and force-driven bone remodelling that contextualise the observed effects of insertion torque and cortical thickness.^4,9,31^ From a biological and clinical standpoint, higher insertion torque and low bone density increase stress at the bone-implant interface, leading to microdamage, impaired blood flow, and reduced implant stability.^6,7,32^ Such excessive mechanical load can also elevate strain energy density within the temporomandibular joint, potentially contributing to adjacent joint or periodontal complications.^23,32^ Smoking further exacerbates these issues by impeding bone healing and intensifying inflammatory responses, particularly in older patients with diminished regenerative capacity.^8,25,34,39^ These risk factors often overlap and compound one another, emphasising the need for individualised treatment planning – especially for patients presenting multiple risk factors. Overall, our findings underscore the importance of meticulous surgical technique, consideration of patient-specific bone characteristics, and a tailored postoperative care plan to enhance both implant longevity and patient comfort.

From a clinical perspective, our findings reinforce the need for a multifaceted approach that addresses both the technical and patient-specific variables impacting orthodontic microimplant success. Clinicians might consider implementing precise insertion torque controls – especially avoiding levels above 15 Ncm – and conducting thorough preoperative assessments for risk factors such as smoking and thin cortical bone. Developing clear decision-making algorithms based on a patient’s age, smoking habits, bone quality, and operator experience can streamline treatment planning and improve outcomes. Furthermore, prioritising patient-centred care by proactively managing postoperative pain is essential for maintaining satisfaction and adherence throughout the orthodontic process. Effective communication regarding potential pain trajectories, along with timely analgesic interventions, can enhance trust, reduce anxiety, and ultimately foster better patient cooperation, underscoring the role of comprehensive and individualised treatment strategies in modern orthodontic practice.

This retrospective, single-centre analysis is vulnerable to selection bias, centre-specific practice patterns, and residual confounding, despite multivariable adjustment. Outcomes were modelled at the implant level, so multiple implants from the same patient may introduce within-patient correlation that can narrow standard errors if unaccounted for. Results should therefore be interpreted as average implantlevel associations. Several variables were extracted from routine records, so insertion torque (documented from charts/torque controllers) and operator experience (binary categorisation) may be subject to measurement simplification. Finally, follow-up for the primary outcome focused on the 0–6-month period, so long-term stability beyond this window could not be evaluated. These design-related issues may affect generalizability, suggesting that prospective, multicenter studies are warranted to confirm causality and extend external validity.

This singlecentre, retrospective analysis identifies practical, modifiable levers for personalised care: (1) control insertion torque and avoid >15 Ncm when feasible; (2) use cortical thickness/bone density to guide site selection and preoperative planning (CBCTinformed in complex cases); (3) provide smokingcessation counselling before placement; and (4) allocate complex or highrisk cases to senior operators and ensure close supervision of trainees. Combined with anticipatory, time-limited analgesia aligned to the observed pain trajectory, these steps translate the present findings (overall success 90.7%; independent effects of smoking, torque, cortical thickness, and operator experience) into a risk-adapted workflow that can improve stability and patient comfort in everyday orthodontic practice. In patients with a reduced or previously diseased periodontium, interdisciplinary planning – SPCsupported orthodontic care and, when indicated, orthodontic management of uneven gingival margins – can be integrated with our riskadapted torque and siteselection strategy to enhance both stability and smile aesthetics.^18,31^

By identifying these risk variables and mapping their influence on postoperative pain – which tends to peak early but can be effectively managed – this study highlights specific intervention points for enhancing patient comfort and implant stability. Together, these insights reinforce the need for careful patient assessment, meticulous torque control, and ongoing operator training in routine clinical practice.

##### Acknowledgements

###### Ethical approval

The study was approved by the Ethics Committee of Qingdao West Coast New Area Central Hospital.

###### Funding

N/A.

###### Conflict of interest

The authors have no conflicts of interest to declare.
